# Transurethral needle electrode resection and transurethral holmium laser resection of bladder cancer

**DOI:** 10.1186/s12957-020-01943-3

**Published:** 2020-07-15

**Authors:** Yu Zhou, Zheng-Long Zhang, Mao-Hua Luo, Hua Yang

**Affiliations:** Department of Urology, Renmin Hospital, Hubei University of Medicine, No. 39 Chaoyang Middle Road, Maojian District, Shiyan, 442000 Hubei China

**Keywords:** Non-muscular invasive bladder cancer, Transurethral needle electrode resection, Transurethral holmium laser resection

## Abstract

**Purpose:**

The aim of the present study was to explore the efficacy and safety of transurethral needle electrode resection and transurethral holmium laser resection of non-muscular invasive bladder cancer (NMIBC).

**Patients and methods:**

In this prospective, case-control study, patients from the Urinary Surgery or Oncology Department who met the inclusion and exclusion criteria received transurethral needle electrode resection (*n* = 52) or transurethral holmium laser resection (*n* = 51).

**Results:**

A total of 103 patients with NMIBC were included in the present study, with 68 males and 35 females. Their mean age was 57.3 years. Sixty-two patients had Ta, 15 patients had T1, and 26 patients had Tis. Operative time, intraoperative blood loss, postoperative gross hematuria time, bladder irrigation time, and postoperative hospitalization time were all significantly lower in the transurethral holmium laser resection group than the transurethral needle electrode resection group. After resection, transurethral holmium laser resection significantly decreased the value of HGF, TSH, and TNF-α versus the transurethral needle electrode resection group. The incidence of obturator reflex was significantly lower in the transurethral holmium laser resection group than the transurethral needle electrode resection group. There was no significant difference in disease-free survival rate and progression-free survival rate between the two groups.

**Conclusions:**

Transurethral holmium laser resection has clinical advantages in the treatment of NMIBC.

## Introduction

Bladder cancer is a common malignancy of the urinary system, and its incidence ranks 9th in the world, 6th in men, and 10th in women. The ratio of male to female incidence is 3:1. According to a 2018 epidemiological report, there were about 78,100 new cases of bladder cancer in China (including about 60,600 cases of male, 17,500 of female), and the incidence is 5.71/10 (8.65/10 of male, 2.62/10 of female). Currently, bladder cancer still carries the highest incidence of urinary reproductive system tumors in China. Pathologically, bladder cancer is divided into muscle- and non-muscle-invasive bladder cancer (NMIBC). NMIBC, which can further develop into muscle-infiltrating bladder cancer, accounts for 70 ~ 80% bladder cancer cases, and 20 ~ 25% of patients with NMIBC have poor prognosis [1–3]. At present, the main problems in clinical practice are insufficient understanding of the risk of NMIBC, which is generally considered to be a low-grade malignancy with a low diagnosis rate of carcinoma in situ (CIS), irregular treatment of special histological types of NMIBC, inadequate treatment of high-risk NMIBC, irregular bladder perfusion treatment, and other problems worthy of attention. The surgical methods of NMIBC include transurethral resection of bladder tumor, photodynamic therapy, transurethral laser surgery, partial cystectomy, and radical cystectomy (RC). RC is divided into three types: open, laparoscopic, and robot-assisted laparoscopic RC. There is substantial evidence that RC is still the most commonly used treatment for high-risk NMIBC or patients who develop muscle-layer infiltration [4, 5].

In this study, we aimed to explore the efficacy of transurethral needle electrode resection and transurethral holmium laser resection for NMIBC based on a prospective case-control study and to provide a basis for the selection of clinical treatment.

## Patients and methods

### Patient selection

This prospective case-control study was conducted from January 2015 to November 2016 at the Department of Urology, Renmin Hospital, Shiyan, China. The study was approved and monitored by the Ethics Committee of Renmin Hospital, Hubei Medical University (approval no. 2017-017). Patients, who were admitted to the Urinary Surgery or Oncology Department, were recruited if they met the following criteria: (1) age ≥ 18 years; (2) pathologically or histologically confirmed primary non-muscular invasive urothelial carcinoma of the bladder (Ta, Tis, T1); (3) Eastern Cooperative Oncology Group (ECOG) performance status (PS) score 0–2; (4) imaging examination showed that bladder cancer did not involve the bladder muscle layer and there was no lymph node metastasis or distant metastasis; (5) subjects who agreed to receive transurethral needle electrode resection or transurethral holmium laser resection and who can be followed up according to postoperative routine perfusion treatment; (6) the function of the main organs (heart, liver, lung, and kidney) was basically normal and ECOG performance status (PS) score 0–2; (7) bladder capacity ≥ 200 mL; and (8) patients provided written informed consent.

Exclusion criteria were as follows: (1) patients diagnosed with muscle-infiltrating urothelial carcinoma of the bladder (stage T2 or above); (2) patients had a history of bladder contracture or bladder functional volume less than 200 mL; (3) severe urethral stricture did not allow entry of a cystoscope; (4) pelvic computed tomography (CT) scan or systemic physical examination revealed tumor metastasis; (5) patients had other concurrent uropoiesis reproductive system tumors (above upper tract urothelial carcinoma, etc.) or tumor in other organs; (6) patients had hereditary or acquired coagulation disorders or were using anticoagulant drugs; (7) significant dysfunction of the main internal organs, such as 1.5 times greater than the upper normal limit of creatinine, aspartate aminotransferase (AST), alanine aminotransferase (ALT), and total bilirubin; (8) decreased hematopoietic function of the bone marrow: leucocytes < 3.0 × 10^9^/L, hemoglobin < 80 g/L, neutrophils < 1.5 × 10^9^/L, platelets < 75 × 10^9^/L, or other hematological diseases; (9) known immunodeficiency, acquired or congenital immunodeficiency, or a history of organ transplantation; (10) metastasis or invasion of the tumor center or various mental disorders; (11) patients had severe cardiovascular and cerebrovascular diseases, such as myocardial infarction, severe/unstable angina pectoris, coronary/peripheral artery bypass graft, symptomatic congestive heart failure, cerebrovascular accident or transient ischemic attack, or pulmonary embolism, who cannot tolerate transurethral resection of bladder tumor (TURBT) surgery; (12) severe hypertension (systolic blood pressure ≥ 180 mmHg, diastolic blood pressure ≥ 110 mmHg), or serious complications of hypertension, or serious complications of diabetes; (13) subjects with fever exceeding 38 °C and significant active infections that may affect the clinical trial, such as acute pneumonia, active tuberculosis, and severe urinary tract infection; (14) those who participated in other clinical trials within 3 months before screening; (15) patients who had received chemotherapeutic drugs or bacille Calmette-Guérin (BCG) perfusion therapy within the last 3 months; (16) severe side effects (symptoms of bladder irritation, etc.) occurred during the treatment of bladder perfusion and the patient was unable to tolerate them; (17) pregnant women, critically ill subjects, and other cases with contraindications, in case of serious cardiovascular disease, significant abnormal coagulation function, non-transitional epithelial tumor (such as adenocarcinoma, squamous cell carcinoma), acute cystitis, spinal deformity and patients cannot lie flat, urethral stricture, etc.; and 18 those who were considered by the researchers to compromise surgical efficacy and safety evaluation or have poor compliance.

This study aimed to explore the efficacy of transurethral needle electrode resection and transurethral holmium laser resection for Non-muscular invasive bladder cancer (NMIBC). The patients were prospectively included in this study and divided into the two groups by the types of surgery.

### Surgical method

#### Transurethral needle electrode resection

The location, number, diameter, and base of the tumor in the bladder were observed by transurethral electrotomy after continuous epidural anesthesia and disinfection. Then, 5% (v/v) mannitol rinse solution was applied to adjust the output power of the electric cutting current to 120 W and the output power of the electric coagulation current to 80 W. The bladder was kept in a filling state, about 1 cm away from the base of the tumor, and the mucosa, submucosa, and deep muscle layer were excised cystoscopically with a needle electrode close to the bladder mucosa. A blunt needle electrode was used to push and peel the deep surface of the deep muscle layer, and the tumor tissues were lifted upward by the impact of water flow, and the tumor base was well exposed. Under the endoscope, tumor tissues and normal tissues could be clearly distinguished. Clockwise or counterclockwise electric cutting was performed along the tumor base until the whole tumor pedicle was completely removed. During the operation, coagulation and cutting were performed to effectively control bleeding and keep the visual field clear. If necessary, the fat in the outer layer of the bladder can be cut. Small tumors were sucked out directly by Elick, while larger tumors were hooked out by a ring-like electrotomy from the operating channel. One tri-lumen indwelling catheter was placed postoperatively, and the bladder was irrigated with normal saline.

#### Transurethral holmium laser resection

After continuous epidural anesthesia and disinfection, a cystoscope was placed through the urethra to observe the location, number, diameter, and base of the tumor in the bladder. A holmium laser fiber was inserted into the bladder through the cystoscope operating hole using normal saline for rinsing. The holmium laser fiber was placed close to the tumor body, and the tumor was cut about 1 cm from the tumor base. When the muscle layer was cut, the tumor tissue was lifted up with water flow. The small tumors were sucked out by Elick directly, while larger tumors were hooked out by a ring-like electrotomy from the operating channel. One tri-lumen indwelling catheter was placed postoperatively, and the bladder was irrigated with normal saline.

### Data collection

Patient baseline information and clinical efficacy data were collected. The baseline information included age, gender, height, weight, body mass index (BMI), tumor size, tumor focality, tumor location, pathological classification, preoperative/postoperative tumor grade, and comorbid disease. The clinical efficacy data included operative time, intraoperative time, postoperative gross hematuria time, bladder irrigation time, postoperative hospitalization time, indwelling time, complications, secondary operation, tumor residual rate at the second operation, disease-free survival rate (1-year, 2-year, 3-year), progression-free survival rate (1-year, 2-year, 3-year), hepatocyte growth factor (HGF), tumor-specific growth factor (TSGF), and tumor necrosis factor alpha (TNF-α). In the follow-up period, B ultrasound and cystoscopy were performed to evaluate recurrence or progress.

### Statistical analysis

Statistical analyses were completed using SPSS17.0. Data were reported as mean (standard deviation) or number (%). Outcomes were compared between two groups with two-sided *t* test for continuous variables and chi-square test for categorical variables. Survival data were analyzed by Kaplan-Meier method. All tests were two-sided, and *P* < 0.05 was considered to be statistically significant.

## Results

### Comparison of baseline characteristics in patients

**Fig. 1:**
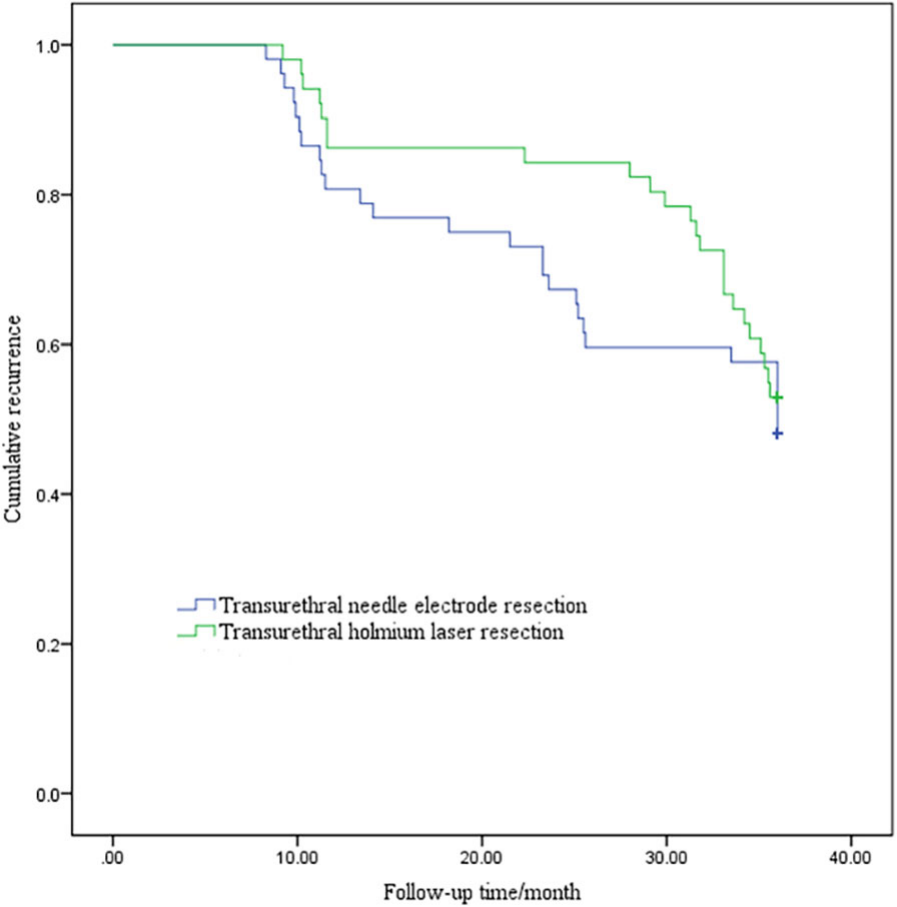
Comparison of tumor recurrence in the follow-up time between the two groups (log-rank test: *χ*^2^ = 0.569, *P* = 0.451)

A total of 103 patients with NMIBC were included in the present study, 52 patients in the transurethral needle electrode resection group, and 51 patients in the transurethral holmium laser resection group (Fig. 1). The patients included 68 males and 35 females, with a mean age of 57.3 years. The pathological classification of NMIBC was as follows: 62 patients in Ta, 15 patients in T1, and 26 patients in Tis. The tumor size was 25.7 (12.6) mm in the transurethral needle electrode resection group, 25.7 (13.9) mm in the transurethral holmium laser resection group. Table 1 shows a comparison of baseline characteristics in patients between the two groups. No statistically significant differences were observed in baseline characteristics of the patients between the two groups (all *P* > 0.05).

**Table 1:** Patients’ basic characteristics of the two surgical groups

Index	Transurethral needle electrode resection (*n* = 52)	Transurethral holmium laser resection (*n* = 51)	Total (*n* = 103)	*P*
Age (year) (mean ± sd)	55.8 ± 12.8	58.8 ± 12.9	57.3 ± 12.9	0.236
Gender [*n* (%)]				
Male	34 (65.4)	3 4(66.7)	68 (66.0)	0.891
Female	18 (34.6)	17 (33.3)	35 (34.0)	
Height (cm) (mean ± sd)	166.6 ± 11.2	163.3 ± 10.0	165.0 ± 10.7	0.120
Weight (kg) (mean ± sd)	64.5 ± 14.9	60.4 ± 12.6	62.4 ± 13.9	0.133
BMI (mean ± sd)	23.0 ± 3.8	22.5 ± 3.5	22.8 ± 3.6	0.487
Tumor size (mm) (mean ± sd)	25.7 ± 12.6	25.7 ± 13.9	25.7 ± 13.2	0.998
Tumor size grouping [*n* (%)]				
< 3 cm	37 (71.2)	33 (64.7)	70 (68.0)	0.483
≥ 3 cm	15 (28.8)	18 (35.3)	33 (32.0)	
Tumor focality [*n* (%)]				
Unifocal	43 (82.7)	43 (84.3)	86 (83.5)	0.825
Multifocal	9 (17.3)	8 (15.7)	17 (16.5)	
Tumor location [*n* (%)]				
Lateral wall	31 (59.6)	31 (60.8)	62 (60.2)	0.993
Trigone	6 (11.5)	5 (9.8)	11 (10.7)	
Dome	7 (13.5)	7 (13.7)	14 (13.6)	
Posterior wall	3 (5.8)	3 (5.9)	6 (5.8)	
Anterior wall	3 (5.8)	2 (3.9)	5 (4.9)	
Less than 1.5 cm from the ureter mouth	2 (3.8)	3 (5.9)	5 (4.9)	
Pathological classification [*n* (%)]				
Ta	33 (63.5)	29 (56.9)	62 (60.2)	0.791
T1	7 (13.5)	8 (15.7)	15 (14.6)	
Tis	12 (23.1)	14 (27.5)	26 (25.2)	
Preoperative tumor grade [*n* (%)]				
G1	44 (84.6)	43 (84.3)	87 (84.5)	1.000
G2	8 (15.4)	7 (13.7)	15 (14.6)	
G3	0 (0.0)	1 (2.0)	1 (1.0)	
Postoperative tumor grade [*n* (%)]				
G1	44 (84.6)	43 (84.3)	87 (84.5)	1.000
G2	7 (13.5)	7 (13.7)	14 (13.6)	
G3	1 (1.9)	1 (2.0)	2 (1.9)	
Comorbid disease [*n* (%)]				
None	30 (57.7)	29 (56.9)	59 (57.3)	1.000
Diabetes	6 (11.5)	6 (11.8)	12 (11.7)	
High blood pressure	8 (15.4)	7 (13.7)	15 (14.6)	
Coronary heart disease (CHD)	4 (7.7)	5 (9.8)	9 (8.7)	
Prostatic hyperplasia	4 (7.7)	4 (7.8)	8 (7.8)	

**Fig. 2:**
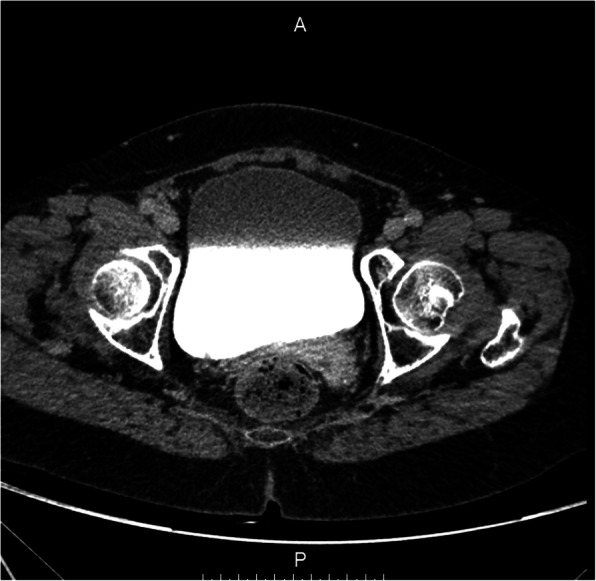
Imaging examination

**Fig. 3:**
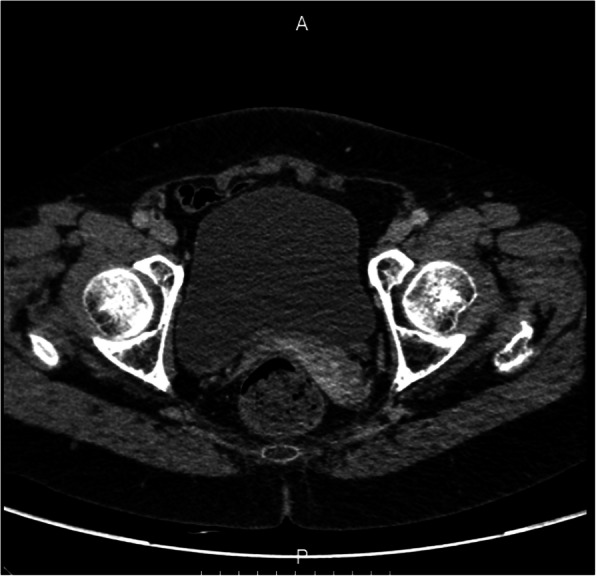
Imaging examination

**Fig. 4:**
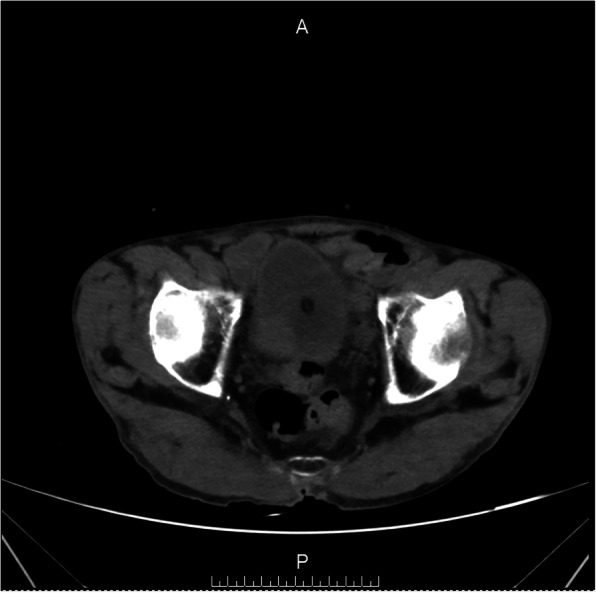
Imaging examination

**Fig. 5:**
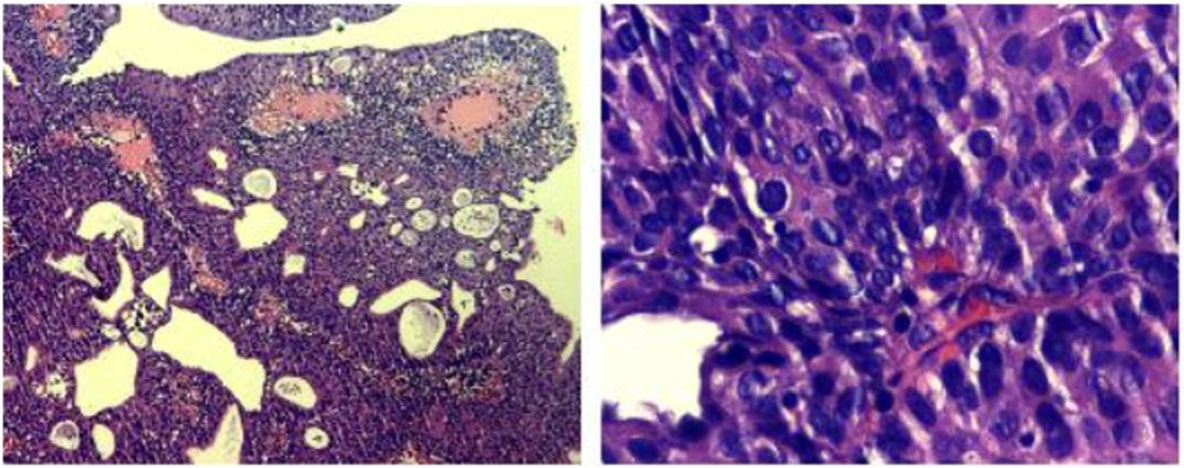
Histopathologic examination

**Fig. 6:**
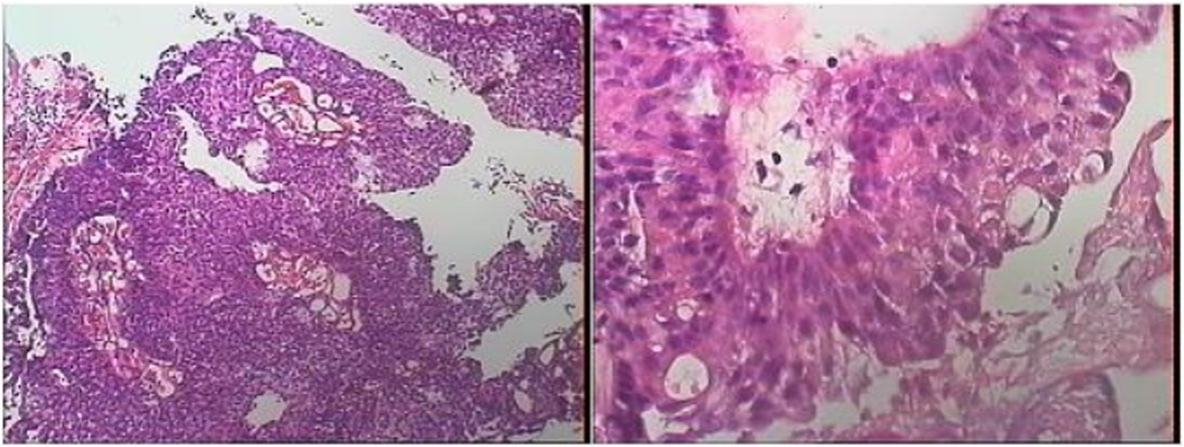
Histopathologic examination

**Fig. 7:**
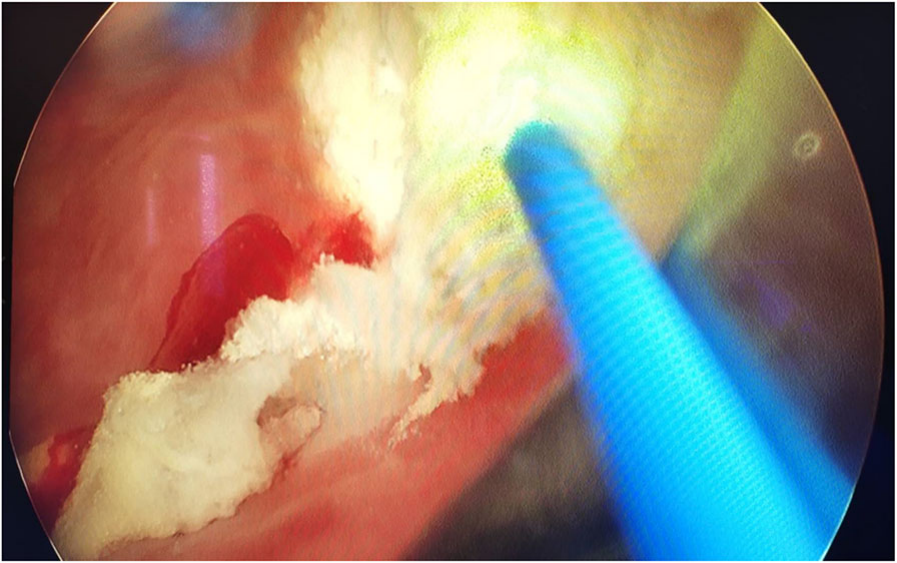
Surgical process

**Fig. 8:**
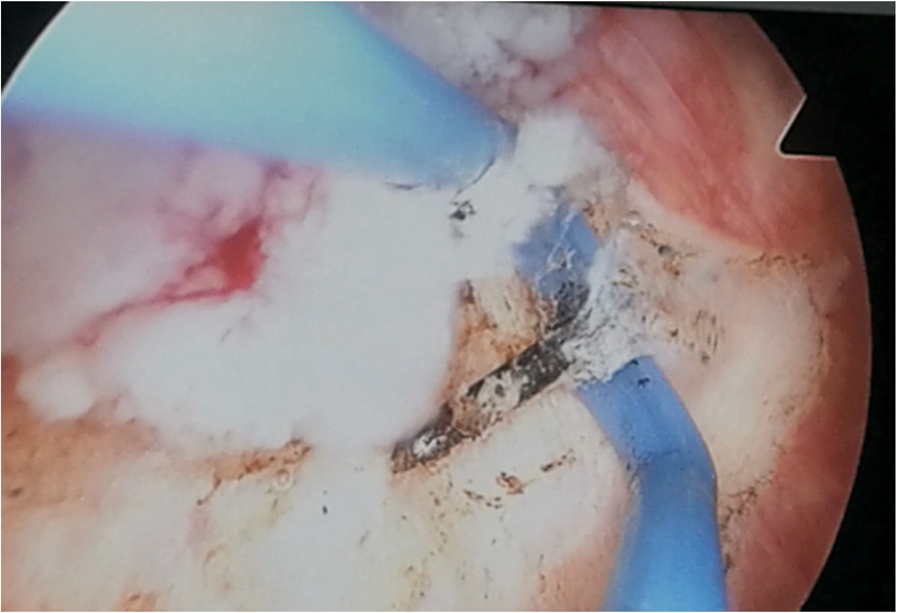
Surgical process

Figures 2, 3, and 4 presented the imaging examination of the bladder tumor on the right posterior wall; Fig. 5 is the histopathologic examination about papillary urothelial carcinoma; Fig. 6 is the histopathologic examination about infiltrating papillary urothelial carcinoma; Fig. 7 is the surgical process about transurethral holmium laser resection; and Fig. 8 is the surgical process about transurethral needle electrode resection.

### Comparison of clinical outcome variables in patients

A comparison was made of the perioperative indicators, complications, and secondary operation after resection between the groups (Table 2). In the transurethral holmium laser resection group, the operative time (28.8 ± 3.6 min vs. 31.5 ± 4.4 min), estimated intraoperative blood loss (27.0 ± 3.9 mL vs. 34.2 ± 6.2 mL), postoperative gross hematuria time (2.8 ± 1.5 days vs. 3.5 ± 1.1 days), bladder irrigation time (1.9 ± 0.5 days vs. 2.2 ± 0.4 days), and postoperative hospitalization time (6.2 ± 1.3 days vs. 6.7 ± 1.3 days) were all significantly decreased after resection when compared with the transurethral needle electrode resection group (*P* < 0.05 or *P* < 0.01). The incidence of obturator reflex was significantly lower in the transurethral holmium laser resection group (2.0%) than the transurethral needle electrode resection group (15.4%) (*P* < 0.05). There was no significant difference in indwelling time, urethral stricture, secondary operation, and tumor residual rate at the second operation between the two groups (all *P* > 0.05).

**Table 2:** Comparison of perioperative indicators between the two surgical groups

Index	Groups		*P*
	Transurethral needle electrode resection (*n* = 52)	Transurethral holmium laser resection (*n* = 51)	
Perioperative indicators (mean ± sd)			
Operative time (min)	31.5 ± 4.4	28.8 ± 3.6	0.001
Intraoperative blood loss (ml)	34.2 ± 6.2	27.0 ± 3.9	0.000
Postoperative gross hematuria time (days)	3.5 ± 1.1	2.8 ± 1.5	0.009
Bladder irrigation time (days)	2.2 ± 0.4	1.9 ± 0.5	0.000
Postoperative hospitalization time (days)	6.7 ± 1.3	6.2 ± 1.3	0.039
Indwelling time (days)	3.4 ± 1.4	3.2 ± 1.0	0.428
Complications [*n* (%)]			
Urethral stricture	3 (5.8)	2 (3.9)	1.000
Obturator reflex	8 (15.4)	1 (2.0)	0.031
Secondary operation [*n* (%)]	14 (26.9)	17 (33.3)	0.478
Tumor residual rate at the second operation [*n* (%)]	3 (21.4)	4 (23.5)	0.889

In the subgroup analysis by tumor size, there was a significant difference in operative time, estimated intraoperative blood loss, and bladder irrigation time between the two groups with tumor size < 3 cm; there was a significant difference in postoperative gross hematuria time between the two groups with tumor size ≥ 3 cm. In the subgroup analysis by tumor focality, there was a significant difference in operative time, estimated intraoperative blood loss, postoperative gross hematuria time, and bladder irrigation time between the two groups with unifocal tumor. In the subgroup analysis by tumor location, there was a significant difference in operative time, estimated intraoperative blood loss, postoperative gross hematuria time, bladder irrigation time, and postoperative hospitalization time between the two groups with lateral wall tumor; there was a significant difference in operative time, postoperative gross hematuria time, and bladder irrigation time between the two groups with posterior wall tumor. In the subgroup analysis by tumor stage, there was a significant difference in operative time, estimated intraoperative blood loss, and bladder irrigation time between the two groups with Ta tumor; there was a significant difference in estimated intraoperative blood loss between the two groups in T1 tumor; and there was a significant difference in estimated intraoperative blood loss and bladder irrigation time between the two groups in Tis tumor.

In the transurethral needle electrode resection group, the 1-, 2-, and 3-year disease-free survival rate was 78.8%, 71.4%, and 64.6%, respectively; the 1-, 2-, and 3-year progression-free survival rate was 73.1%, 61.2%, and 43.8%, respectively. In the transurethral holmium laser resection group, the 1-, 2-, and 3-year disease-free survival rate was 73.1%, 61.2%, and 43.8%, respectively; the 1-, 2-, and 3-year progression-free survival rate was 80.4%, 72.0%, and 54.3%, respectively. There was no significant difference in disease-free survival rate and progression-free survival rate between the two groups (Table 3).

**Table 3:** Comparison of tumor disease-free survival rate and progression-free survival rate of the two surgical groups [*n* (%)]

Group	Disease-free survival rate			Progression-free survival rate		
	1 year	2 years	3 years	1 year	2 years	3 years
Transurethral needle electrode resection	41 (78.8)	35 (71.4)	31 (64.6)	38 (73.1)	30 (61.2)	21 (43.8)
Transurethral holmium laser resection	44 (86.3)	41 (82.0)	34 (73.9)	41 (80.4)	36 (72.0)	25 (54.3)
*P*	0.321	0.213	0.328	0.38	0.255	0.304

There was no significant difference in the perioperative value of HGF, TSH, and TNF-α between the two groups (all *P* > 0.05, Table 4). After the resection, the transurethral holmium laser resection group had a significant decrease in the levels of HGF (10.89 ± 2.47 vs. 16.80 ± 1.45), TSH (2.4 ± 0.4 vs. 3.6 ± 0.3), and TNF-α (76.01 ± 4.82 vs. 99.14 ± 6.01 days) as compared to the transurethral needle electrode resection group (all *P* > 0.05, Table 4).

**Table 4:** Comparison of relevant perioperative and postoperative serum indexes of the two surgical groups

Group	HGF(μmol/L)		TSGF(U/mL)		TNF-α(μg/mL)	
	Preoperative	Postoperative*	Preoperative	Postoperative*	Preoperative	Postoperative*
Transurethral needle electrode resection (*n* = 52)	34.88 ± 4.10	16.80 ± 1.45	5.4 ± 0.8	3.6 ± 0.3	220.33 ± 12.51	99.14 ± 6.01
Transurethral holmium laser resection (*n* = 51)	34.51 ± 4.12	10.89 ± 2.47	5.6 ± 0.7	2.4 ± 0.4	216.75 ± 12.68	76.01 ± 4.82
*P*	0.647	< 0.001	0.153	< 0.001	0.245	< 0.001

*Three days after the operation

## Discussion

Bladder tumors can be divided into two categories: stage Tis, Ta, and T1 non-muscle-infiltrating bladder tumors, and above T2 muscle-infiltrating bladder tumors. NMIBC, also known as superficial bladder tumors, is confined to the mucosa (Ta-Tis) and submucosa (T1) and accounts for about 80 ± 5%. Muscular invasive bladder tumors account for 20 ± 5%. The proportion of Ta, T1, and Tis lesions in NMIBC is about 70%, 20%, and 10%, respectively. According to different recurrence probabilities and prognosis risks, NMIBC can be divided into three levels: low risk, medium risk, and high risk. Risk factors for recurrence are the number of primary tumors, the university, the degree of infiltration and the recurrence probability (especially whether the recurrence time is within 3 months after the surgery), etc., while pathological grade and stage of tumors are associated with tumor progression [6–10].

Traditional TURBT has been widely used in clinical practice, but this method requires the removal of the tumor in sections and layers. During the operation, the tumor is morselized and removed. This method violates the principle of no tumor in traditional surgery and may lead to tumor cell dispersion and implantation, increasing the recurrence rate of bladder tumor after surgery. Secondly, this kind of operation inevitably destroys the tumor layer, and the specimen may miss the muscle layer, resulting in the inaccuracy of postoperative pathological staging, thus affecting subsequent treatment. In addition, some specific sites, such as tumors located near the lateral posterior wall of the bladder, are prone to obturator nerve reflex during surgery because the pelvic segment of the obturator nerve travels along the lateral wall of the pelvis and is close to the lateral posterior wall of the bladder [11–16].

Currently, the treatment options of NMIBC include surgical treatment, postoperative cystic-perfusion chemotherapy, and cystic-perfusion immunotherapy. The surgical treatment includes transurethral resection of bladder tumor, transurethral laser surgery, and photodynamic therapy. TURBT is the current “gold standard” for the treatment of NMIBC and also the main treatment [17–19]. The objectives of TURBT are as follows: first, complete removal of all tumors within the field of vision to achieve the effect of radical surgical resection; second, resection of tissues for evaluation of pathological grade and stage, determination of the next postoperative treatment plan of the patient, and assessment of prognosis and risk of recurrence. In 1966, lasers began to be used in urology. This technique has been widely used in clinical practice for its advantages of safety, simplicity, less damage, and definite curative effect. In recent years, holmium laser, green laser, and thulium laser have been developed and put into clinical use, showing a good clinical application prospect. Holmium laser was used to treat bladder tumors in the mid-1990s and has been widely used because of its good cutting and electrocoagulation, shallow tissue penetration, and low heat loss [20–23].

TURBT is the preferred surgical method for the treatment of NMIBC at present. It mainly relies on electrothermal effect to remove tumors. Due to the physical characteristics of electrotomy, this surgical method has some unavoidable defects. The TURBT circuit current can easily induce obturator nerve reflex during tumor resection of the lateral wall of the bladder. Strong obturator nerve reflex can cause complications such as bladder perforation. If the tumor location is unique, risks such as ureteral orifice stricture, urethral stricture, tumor residue, and disseminated implantation may occur after TURBT, as well as disadvantages such as easy burning of tumor specimens to form eschar and difficulty in accurate pathological staging [24–27].

Holmium laser is a pulsed solid-state laser, which generates strong heat in an instant and can be absorbed by superficial tissues efficiently. Its tissue penetration depth is 4 mm, and it has good vaporization cutting and coagulation hemostatic ability, which can precisely cut tumor tissues to effectively avoid bladder perforation. In addition, the holmium laser does not generate an electric field during surgery and can completely eliminate the obturator nerve reflex. It is suitable for patients with cardiac pacemaker and arrhythmia. It is worth noting that the temperature of the local tissue can be as high as 100 ~ 300 °C [28]. In contrast, the local tissue temperature during laser treatment of NMIBC is only 40 ~ 75 °C, and the thermal damage of the surrounding tissues is relatively small. Therefore, postoperative wound healing is fast and the indwelling catheter time is short.

In our study, the operative time, estimated intraoperative blood loss, postoperative gross hematuria time, bladder irrigation time, and postoperative hospitalization time were all significantly lower in the transurethral holmium laser resection group as compared to the transurethral needle electrode resection group. After resection, transurethral holmium laser resection significantly decreased the value HGF, TSH, and TNF-α as compared to transurethral needle electrode resection. The incidence of obturator reflex was significantly lower in the transurethral holmium laser resection group than the transurethral needle electrode resection group.

## Conclusion

In the treatment of NMIBC, holmium laser resection has the advantages of fewer complications, better safety, faster postoperative recovery, and lower recurrence rate versus traditional transurethral needle electrode resection. The limitations of this study include the small number of cases and lack of randomized controlled study with TURBT, so it is still necessary to carry out a large-scale randomized controlled clinical study with long-term follow-up to verify its long-term therapeutic effect.

## Data Availability

The datasets during and/or analyzed during the current study are available from the corresponding author on reasonable request.

## Abbreviations

CIS: Carcinoma in situ
HGF: Hepatocyte growth factor
NMIBC: Non-muscular invasive bladder cancer
RC: Radical cystectomy
TNF-α: Tumor necrosis factor alpha
TSGF: Tumor-specific growth factor
RC: Radical cystectomy
ECOG: Eastern Cooperative Oncology Group
PS: Performance status
CT: Computed tomography
AST: Aspartate aminotransferase
ALT: Alanine aminotransferase
TURBT: Transurethral resection of bladder tumor
BCG: Bacille Calmette-Guérin
BMI: Body mass index
